# TRENTOOL: an open source toolbox to estimate neural directed interactions with transfer entropy

**DOI:** 10.1186/1471-2202-12-S1-P200

**Published:** 2011-07-18

**Authors:** Michael Wibral, Raul Vicente, Viola Priesemann, Michael Lindner

**Affiliations:** 1 MEG Unit, Brain Imaging Center, Goethe University, Frankfurt, Germany; 2 Frankfurt Institute for Advanced Studies, Goethe University, Frankfurt, Germany; 3 Dept. Neurophysiology, Max Planck Institute for Brain Research, Frankfurt, Germany; 4 Neural Systems and Coding, Max Planck Institute for Brain Research, Frankfurt, Germany; 5 Group for Neural Theory, Ecole Normale Superieure, Paris, France; 6 Center for Economics and Neuroscience, University Bonn, Bonn, Germany

## 

To investigate directed interactions in neural networks we often use Norbert Wiener's famous definition of observational causality. Wiener’s definition states that an improvement of the prediction of the future of a time series X from its own past by the incorporation of information from the past of a second time series Y is seen as an indication of a causal interaction from Y to X. Early implementations of Wiener's principle – such as Granger causality – modelled interacting systems by linear autoregressive processes and the interactions themselves were also assumed to be linear. However, in complex systems – such as the brain – nonlinear behaviour of its parts and nonlinear interactions between them have to be expected. In fact nonlinear power-to-power or phase-to-power interactions between frequencies are reported frequently. To cover all types of non-linear interactions in the brain, and thereby to fully chart the neural networks of interest, it is useful to implement Wiener's principle in a way that is free of a model of the interaction [1]. Indeed, it is possible to reformulate Wiener's principle based on information theoretic quantities to obtain the desired model-freeness. The resulting measure was originally formulated by Schreiber [2] and termed transfer entropy (TE). Shortly after its publication transfer entropy found applications to neurophysiological data. With the introduction of new, data efficient estimators (e.g. [3]) TE has experienced a rapid surge of interest (e.g. [4]). Applications of TE in neuroscience range from recordings in cultured neuronal populations to functional magnetic resonanace imaging (fMRI) signals. Despite widespread interest in TE, no publicly available toolbox exists that guides the user through the difficulties of this powerful technique. TRENTOOL (the TRansfer ENtropy TOOLbox) fills this gap for the neurosciences by bundling data efficient estimation algorithms with the necessary parameter estimation routines and nonparametric statistical testing procedures for comparison to surrogate data or between experimental conditions. TRENTOOL is an open source MATLAB toolbox based on the Fieldtrip data format.

We evaluated the performance of the toolbox on simulation data and also a neuronal dataset that provides connections that are truly unidirectional to circumvent the following generic problem: typically, for any result of an analysis of directed interactions in the brain there will be a plausible explanation because of the combination of feedforward and feedback connectivity between any two measurement sites. Therefore, we estimated TE between the electroretinogram (ERG) and the LFP response in the tectum of the turtle (Chrysemys scripta elegans) under visual stimulation by random light pulses. In addition, we also investigated transfer entropy between the input to the light source (TTL pulse) and the ERG, to test the ability of TE to detect directed interactions between signals with vastly different properties. We found significant (p<0.0005) causal interactions from the TTL pulse to the ERG and from the ERG to the tectum – as expected. No significant TE was detected in the reverse direction.

## CONCLUSION

TRENTOOL is an easy to use implementation of transfer entropy estimation combined with statistical testing routines suitable for the analysis of directed interactions in neuronal data.
